# Internet of things driven hybrid neuro-fuzzy deep learning building energy management system for cost and schedule optimization

**DOI:** 10.3389/frai.2025.1544183

**Published:** 2025-03-26

**Authors:** Deepshikha Shrivastava, Prerna Goswami

**Affiliations:** ^1^Applied Science Department-Electrical, Pimpri Chinchwad College of Engineering and Research, Pune, India; ^2^General Engineering Department-Electrical, Institute of Chemical Technology, Mumbai, India

**Keywords:** electricity disaggregation, internet of energy, energy consumption monitoring, DL, building energy management system

## Abstract

Optimizing building energy consumption holds significant untapped potential, particularly in a developing economy such as India. Existing solutions have yet to concentrate on a methodology that is cost-effective, small-scale, precise, and open source data-driven. In response, we have implemented an automated, DL-enabled approach to predict energy consumption with the goal to enable cost and schedule optimization. For two years from December 2021 to December 2023 the energy consumption and twenty seven associated energy parameters was monitored by developing an IoT enabled BEMS. The data collected was preprocessed, cleaned, transformed and used for training a machine learning model. Based on the previous literature, a hybrid DL model was developed using artificial neural networks and fuzzy logic by integrating fuzzy layers in the deep neural architecture. The collected electrical data was used for training, hyper-parameter tuning and testing the hybrid DL model. The proposed model when tested for out-of-sample dataset had comparable results on error and performance metrics as compared to other states of the art models. On deployment in the premises of a university, the BEMS achieved a reduction in the electricity bill of 20% highlighting its effectiveness and efficacy.

## 1 Introduction

Buildings account for 33% of the final energy usage and contributing to 40% of both direct and indirect CO_2_ emissions worldwide (Nabavi et al., 2021). This makes monitoring electricity consumption and the cost and task optimization, a crucial focus area in energy management and conservation (Abu-Shikhah et al., 2011). Cost and task optimization involves organizing the high energy consuming activities during off-peak hours. It also involves fixing the voltage, current, apparent power and phase parameters to their optimal values to achieve energy savings. Numerous studies have investigated the implementation of Arduino Uno for monitoring electricity consumption for cost and task optimization (Adepoju et al., 2007). There has been a notable increase in interest toward employing data science along with machine learning (ML) and DL techniques, including supervised and unsupervised methodologies, to optimize consumption patterns of micro, small, and medium enterprises (MSME) (Liu et al., 2019). Electricity optimization systems are advantageous because they deliver real-time data on energy usage, thereby enhancing energy efficiency and reducing costs. Predominantly, low-cost Arduino-based systems are utilized to incorporate energy sensors to measure electrical appliances’ current and voltage (Al-Anbuky and Bataineh, 1995). These sensors relay data to the Arduino Uno, which then computes power consumption and transmits the relevant information to a centralized monitoring unit (Moutaib et al., 2022). However, the primary limitations are associated with the Arduino Uno’s lifespan, heat tolerance, lack of wireless capabilities, and limited memory and processing power. These factors constrain its widespread adoption at the MSME level (Amjady, 2002).

Nevertheless, certain impediments obstruct research progress in this domain. These include the protracted nature of ground-level data collection (sometimes extending up to multiple years), restricted access to enterprise infrastructure due to privacy and security issues, the initial investment in hardware (routers, microcontrollers, modems, gateways and cloud services), the unpredictability of research results, and the requirement for ongoing monitoring. Consequently, research groups often exhibit reluctance to undertake studies in the electricity disaggregation field.

Most research in this area focuses on preventing electricity theft, with a few significant exceptions targeting energy consumption optimization in wireless sensor networks (WSN) (Elashry et al., 2024). Various methodologies are deployed for predictive tasks across different fields. These methodologies are generally evaluated on the basis of two main criteria: implementation ease and result accuracy. They can be broadly categorized into mathematical and artificial intelligence-based methods. Mathematical approaches include techniques such as regression analysis and exponential smoothing [Madhav and Sunil, 2022], while artificial intelligence methods encompass ANNs, knowledge-based expert systems, and fuzzy logic (Okolobah and Ismail, 2013). One approach to boost predictive accuracy is to combine multiple methodologies, leveraging the strengths of each to counterbalance any weaknesses (Montgomery et al., 1990). The effective application of these predictive methods crucially depends on the availability of historical data, which forms the foundation for forecasting future trends (Liu et al., 2019).

The following are the primary contributions of this paper: the research involves data collection from the Pimpri Chinchwad College of Engineering and Research (PCCoER), Pune, India, over a two-year surveillance period. The collected data includes various characteristics not previously integrated at this scale into predictive analytics. The methods’ effectiveness was assessed using statistical metrics (Yildiz et al., 2017; Saravanan et al., 2012).

- Collect and preprocess electricity consumption data from a university campus over 2 years. Data was collected continuously 24/7 at one-minute intervals, recording several parameters related to electricity consumption in a cloud database. These parameters, identified through existing literature, include energy, frequency, power, apparent power, current, and volt-ampere hours. To the best of our knowledge, this is the most extensive data collection effort in a university setting in India.
- Analyze the data to discover patterns and trends in electricity consumption throughout the day. The intent is to understand the relationships between the parameters and validate their theoretical connections. Moreover, preprocessing will identify data collection anomalies and apply necessary data transformations.
- Develop DL models to predict electricity consumption based on the time of day, weather conditions, and other pertinent factors. These models aim to determine which independent variables are most predictive of the dependent variable, energy consumption. The most suitable DL model is selected based on evaluation against other state-of-the-art models.
- Implement cost and load optimization strategies based on the DL models with a view to reduce the energy costs of the infrastructure by scheduling high energy consuming activities during off-peak hours. Assess the effectiveness of optimized strategies in reducing energy consumption and costs.

Following an extensive literature review, this work is among the first practical studies on electricity consumption using an IoT setup in Indian context (Adepoju et al., 2007; Amjady, 2002). The experiments and data collection were conducted using open-source tools and costeffective hardware, making it feasible for shop floor deployment by interested parties. While there have been efforts in Western and Southeast Asian countries to map energy consumption and optimize cost and load, our work differs from these studies. Further, the ever-evolving technological landscape, necessitates an updated study in this domain.

The paper is organized as follows: Section 2 provides an overview of the previous literature and state-of-the-art in IoT-based automated electricity consumption monitoring. Section 3 details the data collection, mathematical model, data preprocessing, feature description, and experimental setup. The experimental results are discussed in Section 4, followed by the conclusion and future research directions in Section 5.

## 2 Related works

The review explores the advantages and disadvantages of current research in the field of electricity consumption reducing using cost and load optimization. The papers covers non-intrusive and intrusive consumption monitoring at various levels, including building, grid, and cloud environments in this section. Both non-intrusive and intrusive approaches offer different benefits and drawbacks. Intrusive load monitoring methods require numerous dedicated sensors installed on individual appliances to gather their power consumption data. In contrast, non-intrusive load monitoring (NILM) necessitates a single measurement at the main power service entry, effectively allowing for power load monitoring and identification with fewer resources (Yang et al., 2020). The key benefit of intrusive systems is their device-level measurement precision, whereas NILM boasts lower installation and maintenance costs and better consumer acceptance in terms of privacy and convenience.

Kim et al. (2019) aimed to detect non-technical losses resulting from meter manipulation or malfunctioning by leveraging Arduino Uno for monitoring electric consumption in residential buildings. They built a smart metering system using Arduino Uno and validated it in a residential setting. The system accurately measured and monitored different household appliances’ electricity consumption. However, implementing the system demands substantial investment due to its multi-tier architecture. Ibrahem et al. (2020) made a similar attempt at load monitoring to mitigate electricity theft by using Arduino Uno in commercial buildings. They developed a system integrating Arduino Uno with energy meters to track and analyze electricity use across various devices. This approach also entails a multi-tier architecture, making it costly and less suitable for small or medium-sized businesses. Both studies (Kim et al., 2019; Ibrahem et al., 2020) emphasize the significance of employing data science techniques like ML and DL for identifying trends in temporal data.

While not directly related to energy consumption monitoring in MSMEs, Bicici (2024) introduced an application called Cloud Monitor aimed at reducing cloud environment energy consumption. The author proposed a linear programming optimization strategy for managing cloud loads to decrease energy use, although the results were derived from simulations rather than real-world environments. Hannan et al. (2018) conducted a comprehensive review of BEMS and discussed challenges like data loss and network issues. They advocated for BEMS that are energy-efficient, cost-effective, easy to install, and tamper-proof—all critical factors considered in the current research architecture.

Researchers have conflicting opinions on the impact of BEMS on energy consumption. Previous studies have shown feedback can drive short-term energy savings, but Alahmad et al. (2011) found in a US survey that BEMS feedback did not significantly translate to energy savings. Martınez-Sanchez et al. (2023) demonstrated how energy savings can enhance profitability for an MSME and contribute to sustainable development goals (SDGs). Given these findings, we chose PCCoER as our target institution where energy monitoring was previously non-existent. Savings in energy consumption here would directly benefit students via reduced tuition fees. Our data-driven approach is similar to the work of Kang et al. (2020), with the main difference being our focus on BEMS, whereas they focused on specific machines. The methodology proposed by Zhuang et al. (2024) for data collection, preprocessing, and ML pipeline for electricity consumption forecasting was also influential. Additionally, Zhang et al. (2024) proposed a cloud-fog cooperation scheduling algorithm to optimize energy use and delay functions in cloud environments.

We reviewed approaches and technologies suggested for energy grid monitoring (Atalik et al., 2013; Blair et al., 2016). Although these methods are designed for larger infrastructures and not directly applicable to BEMS, the power quality parameters highlighted were beneficial for our feature engineering. Our work emphasizes BEMS exclusively, thus excluding issues of security and malware attacks, which are covered in the work of (Jiang et al., 2018). For those interested in case studies on electricity consumption monitoring in smart grids, Janthong et al. (2023) provides a relevant case study.

The studies collectively indicate the significant role of DL in data analysis and inference. The limitations of existing methods can be summarized as follows:

- High cost and scarce open-source techniques
- Limited applicability to small-scale buildings
- Inadequate use of data-driven methodologies

Neuro-fuzzy systems, an evolutionary integration of neural networks and fuzzy logic, bring a unique approach to the ML/DL landscape. These systems utilize the learning capabilities of neural networks and the human-like reasoning style of fuzzy logic. Neuro-fuzzy models, such as Adaptive Neuro-Fuzzy Inference Systems (ANFIS), are advantageous for handling uncertainty and imprecise information, offering good interpretability and robustness.

However, neuro-fuzzy systems have their own limitations. They can become computationally expensive, especially as the complexity of the fuzzy rule base increases. Additionally, creating an optimal fuzzy rule set and membership functions often requires expert domain knowledge, making them less flexible than purely data-driven approaches like deep learning. Despite these drawbacks, neuro-fuzzy systems play a crucial role in applications where interpretability and dealing with ambiguity are essential, situating themselves effectively in the broader ML/DL ecosystem.

## 3 Proposed hybrid DL-IoT BEMS

**Figure 1:**
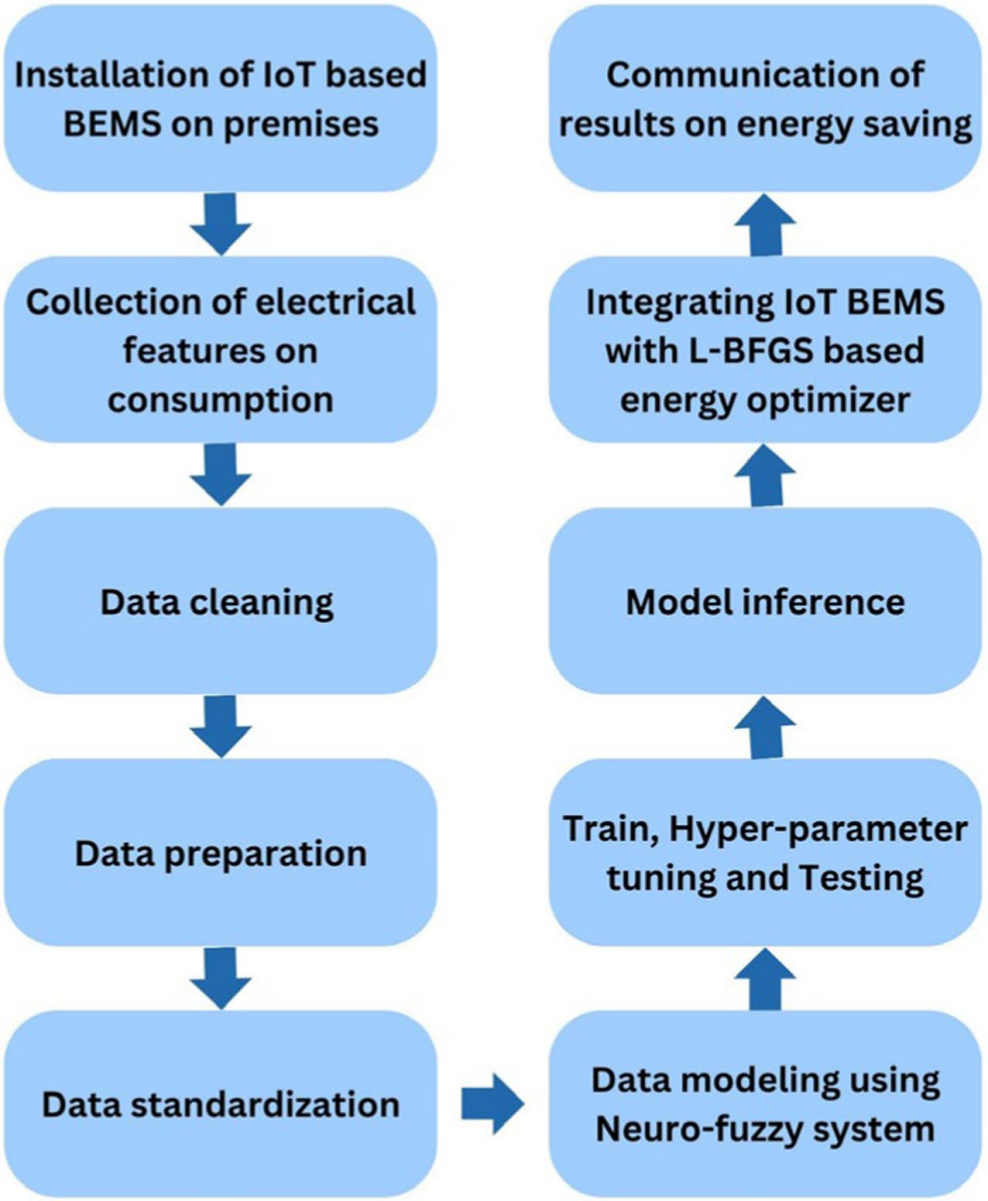
IoT BEMS workflow.

**Figure 2:**
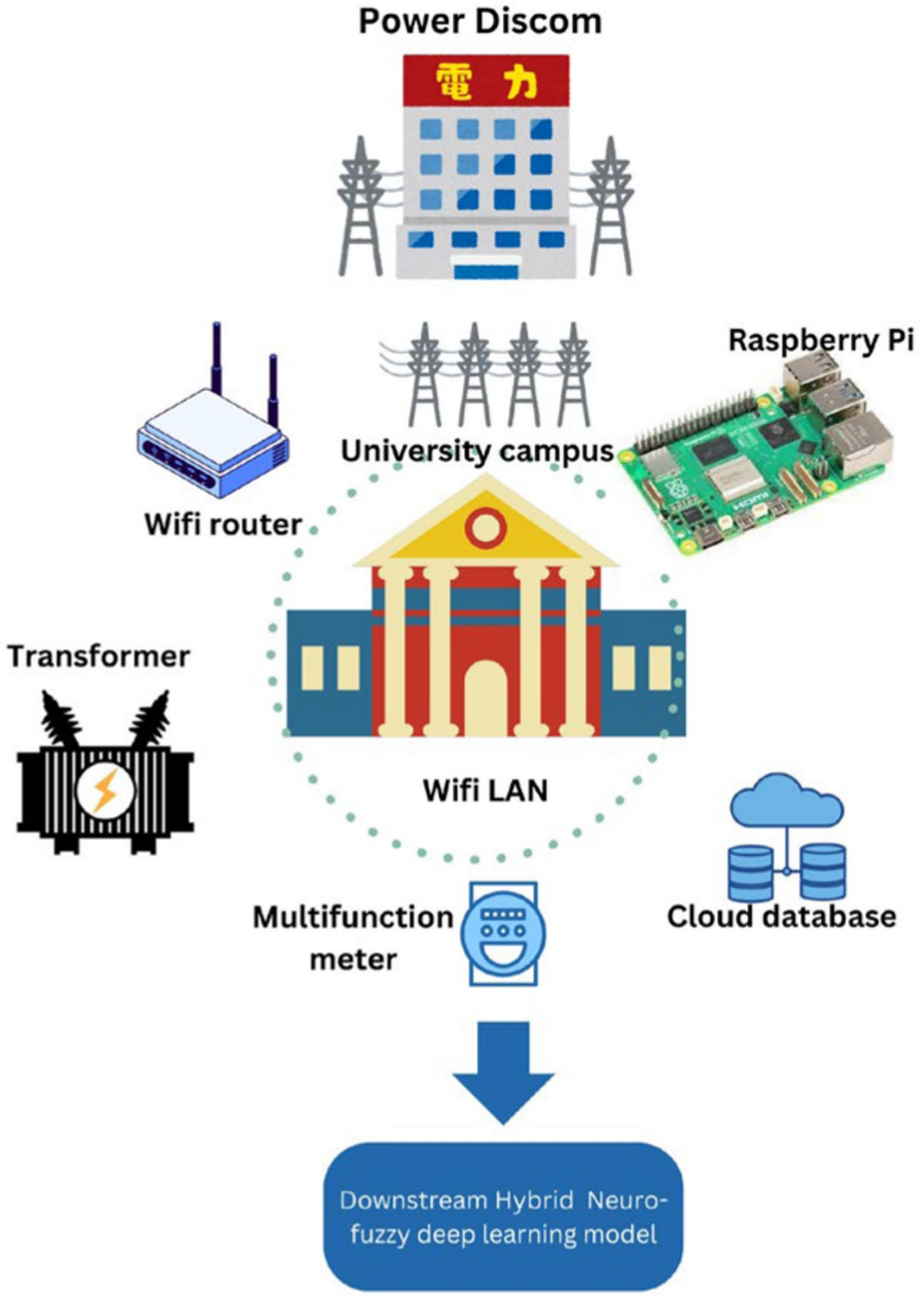
System architecture for DL based BEMS.

The workflow of the procedure is the illustrated in Figure 1 with all the steps for implementation of the IoT BEMS. This study presents a structured workflow for an IoT-based Building Energy Management System (BEMS) with an integrated energy optimization mechanism utilizing a Neuro-fuzzy system and L-BFGS optimization. The proposed framework encompasses data acquisition, preprocessing, modeling, and optimization, leading to enhanced energy efficiency in smart buildings. The study aims to improve energy consumption patterns through predictive modeling and real-time optimization (Figure 2).

### 3.1 Data collection

To monitor electricity consumption, a comprehensive data collection setup was established, which included the installation of a multifunction meter, a Raspberry Pi 3B, a USB connector, a GSM (WiFi) modem, and a current transformer (Figures 3–10).

**Figure 3:**
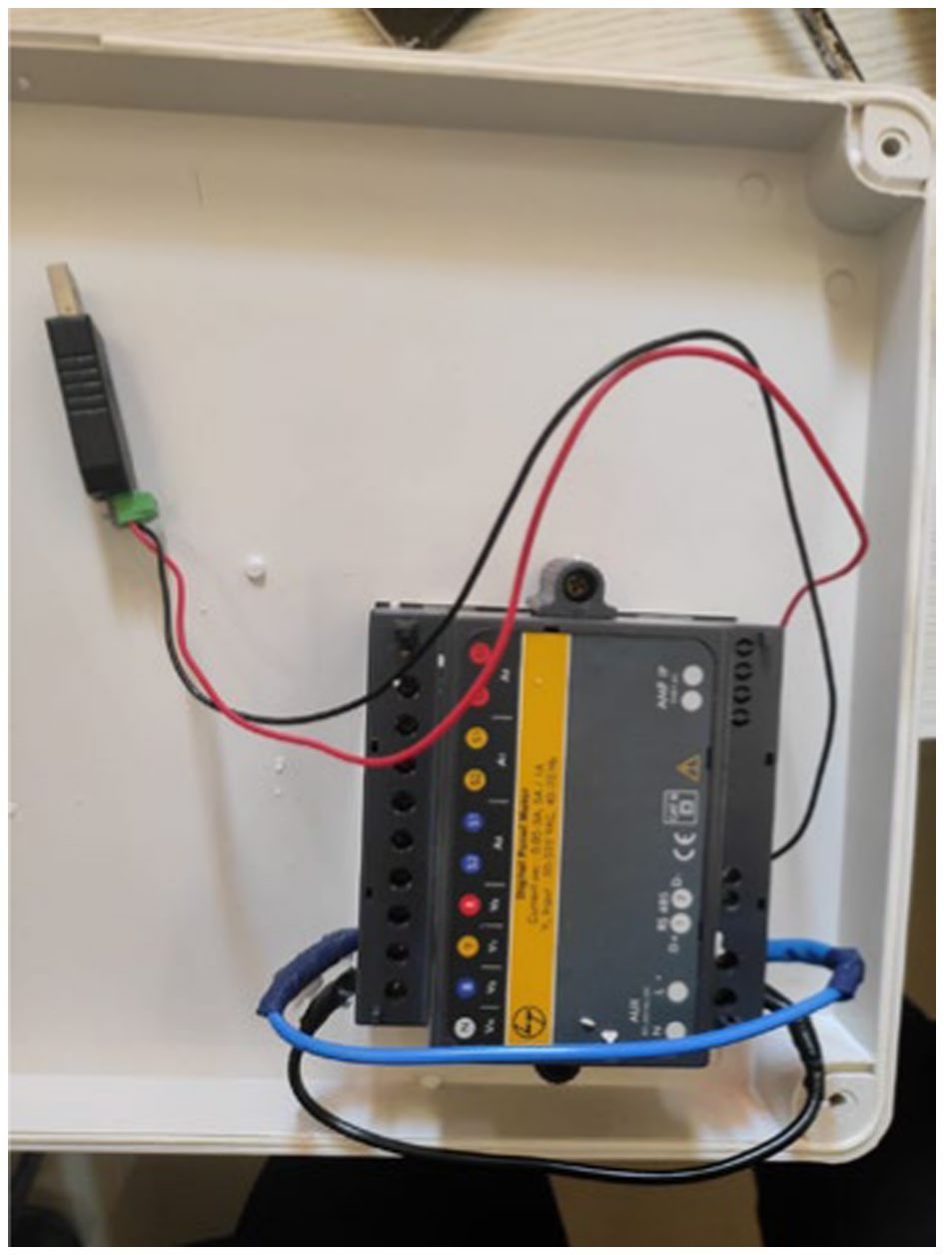
Multifunction meter.

**Figure 4:**
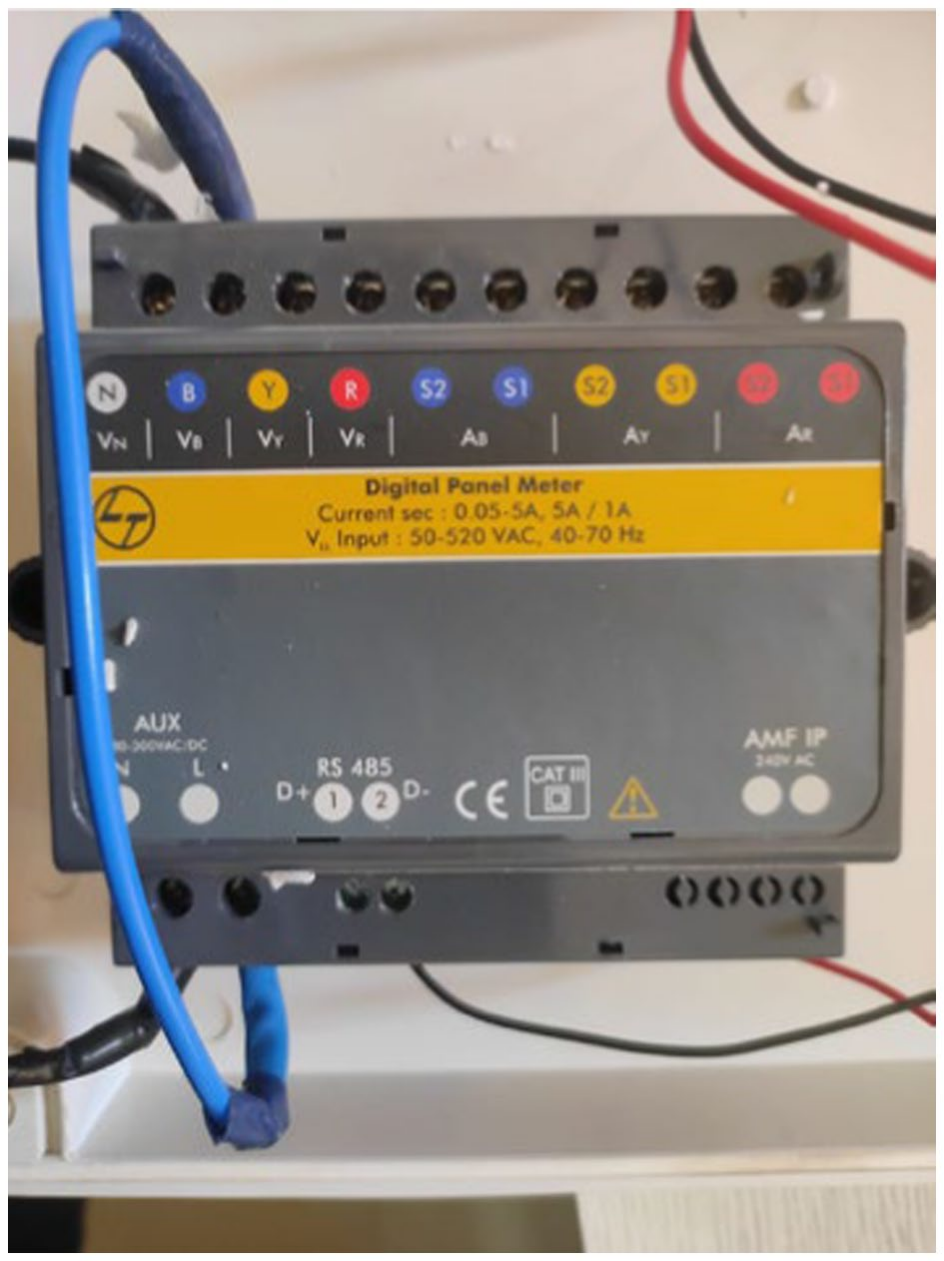
Multifunction meter.

**Figure 5:**
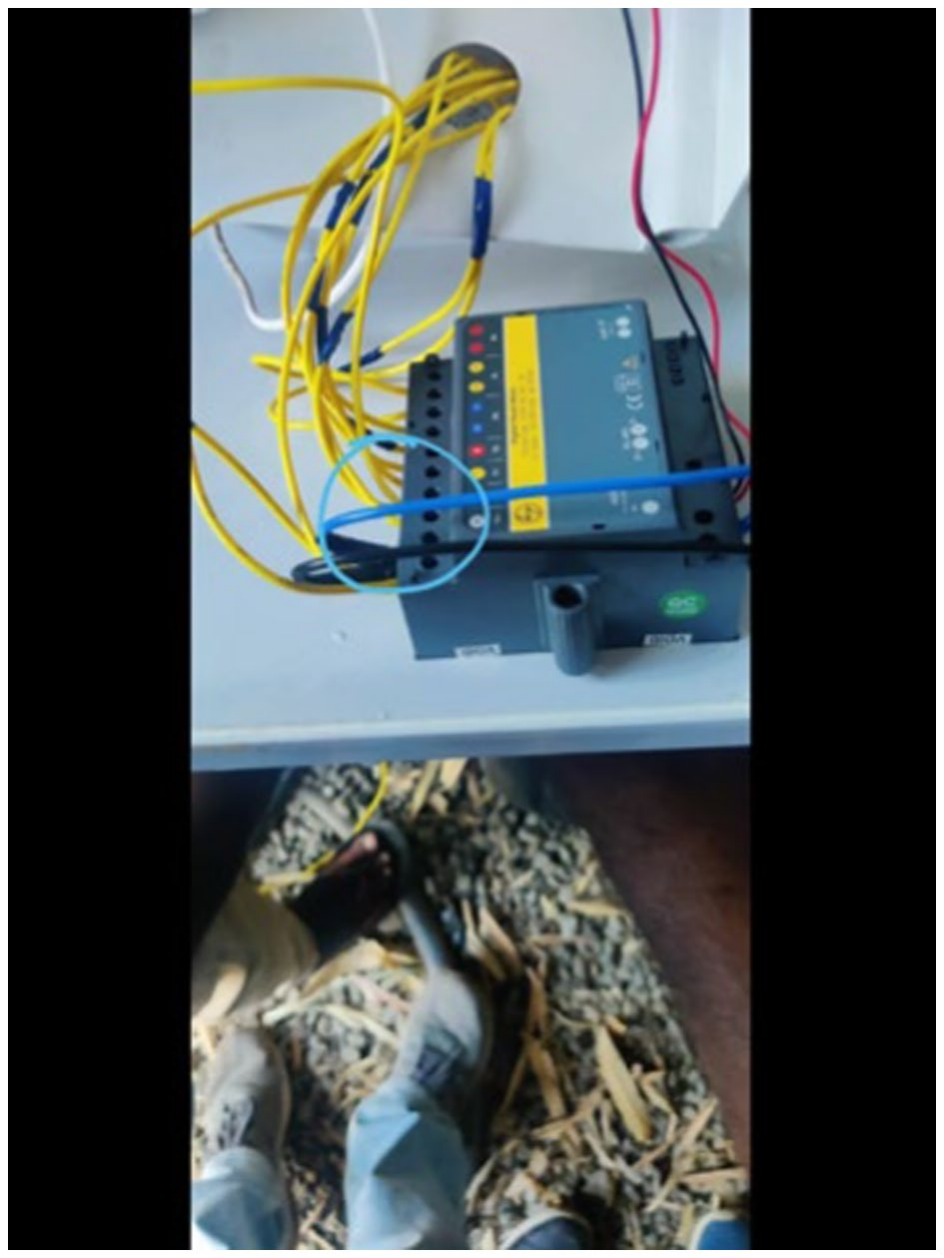
Wired connection multifunction meter.

**Figure 6:**
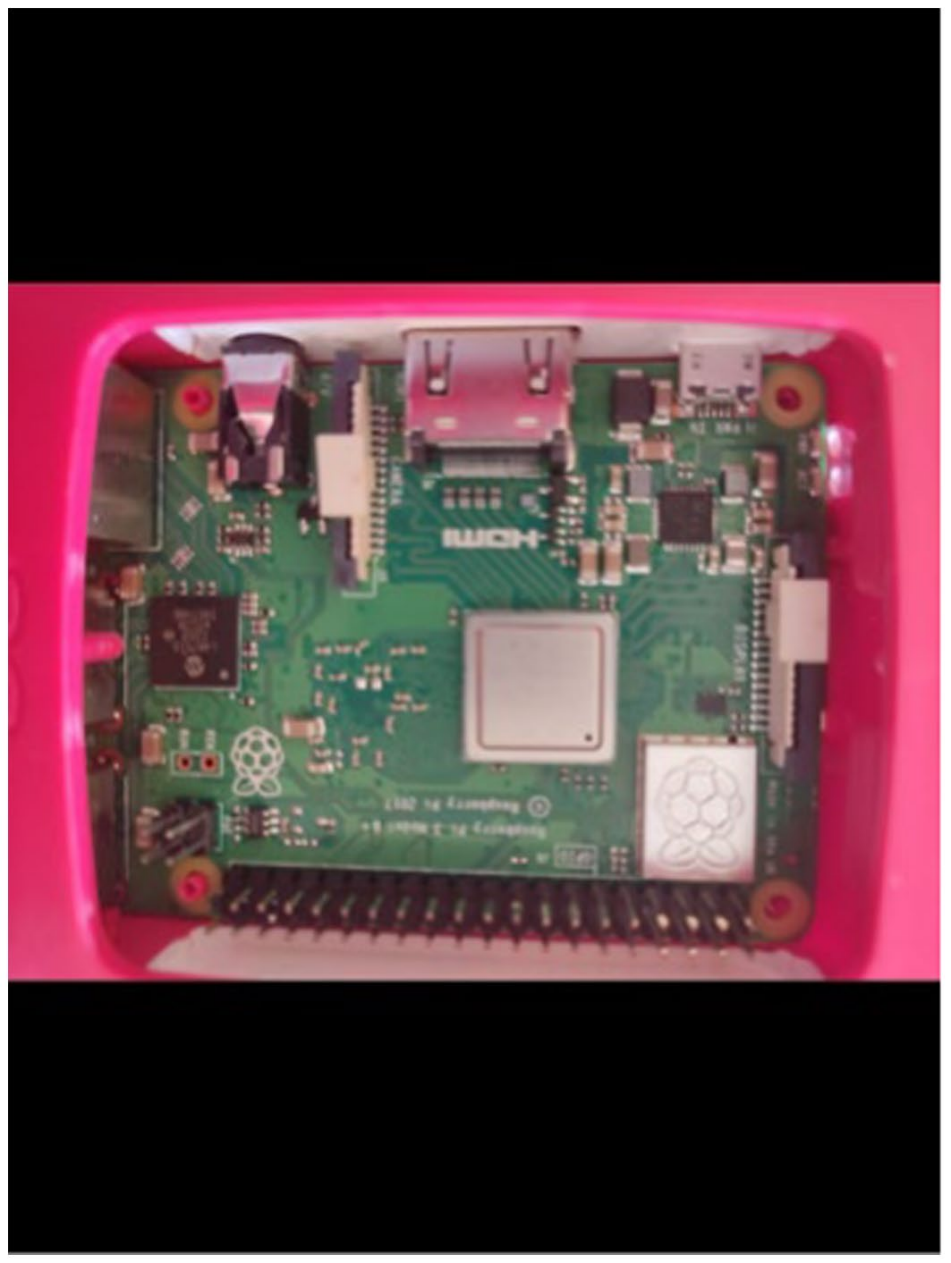
Raspberry pi.

**Figure 7:**
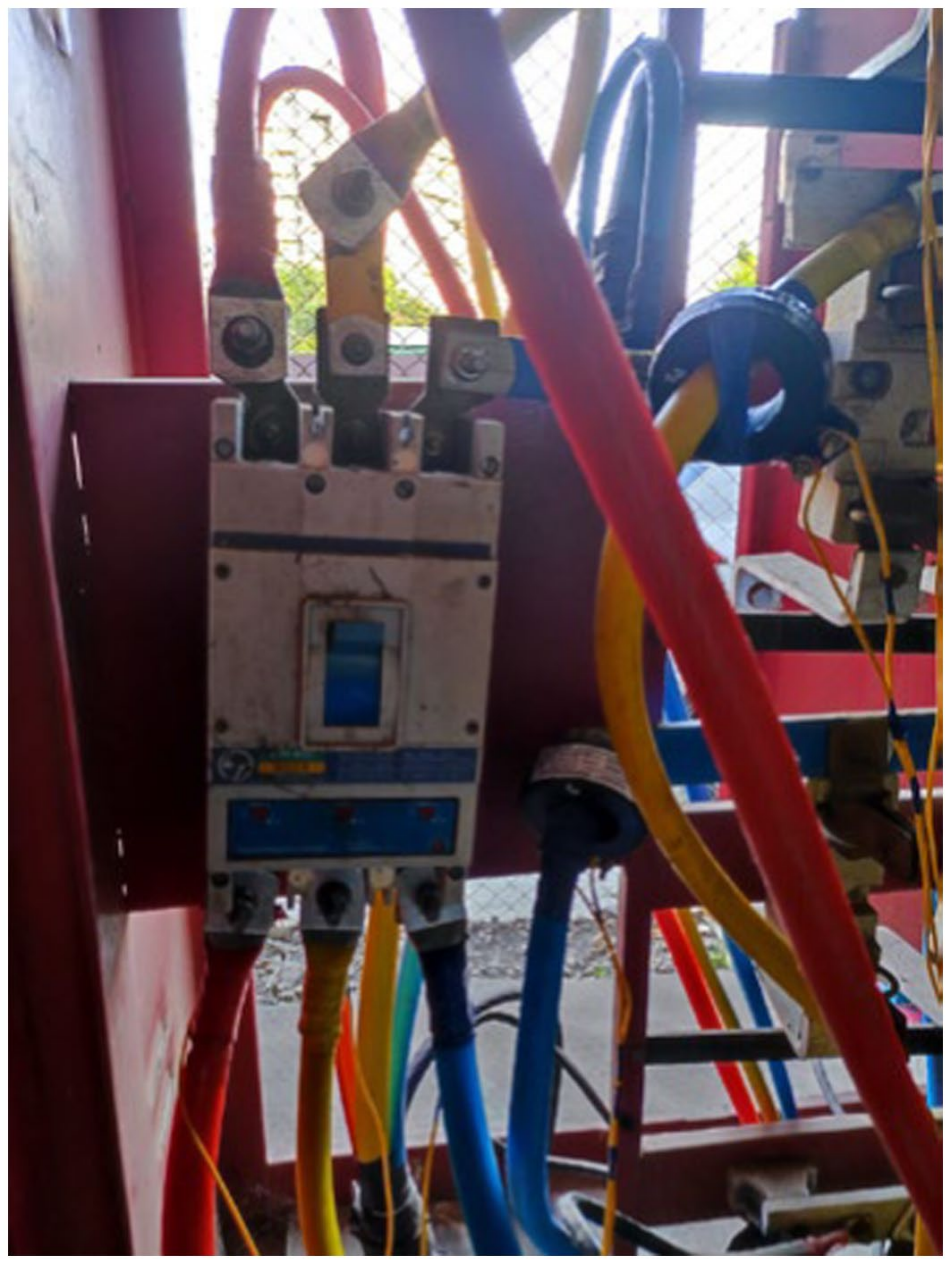
Current transformer to main line.

**Figure 8:**
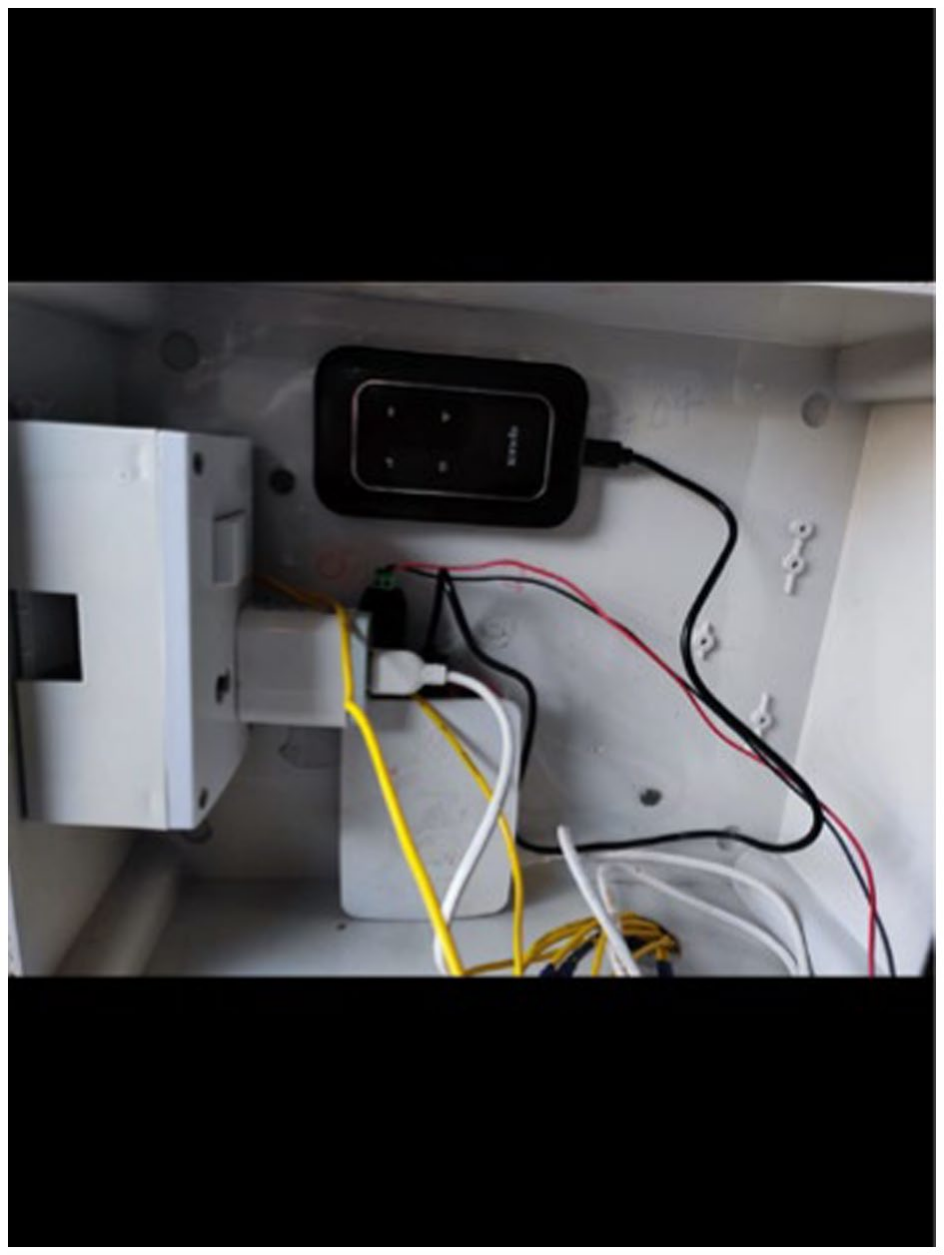
USB connector and Wifi modem.

**Figure 9:**
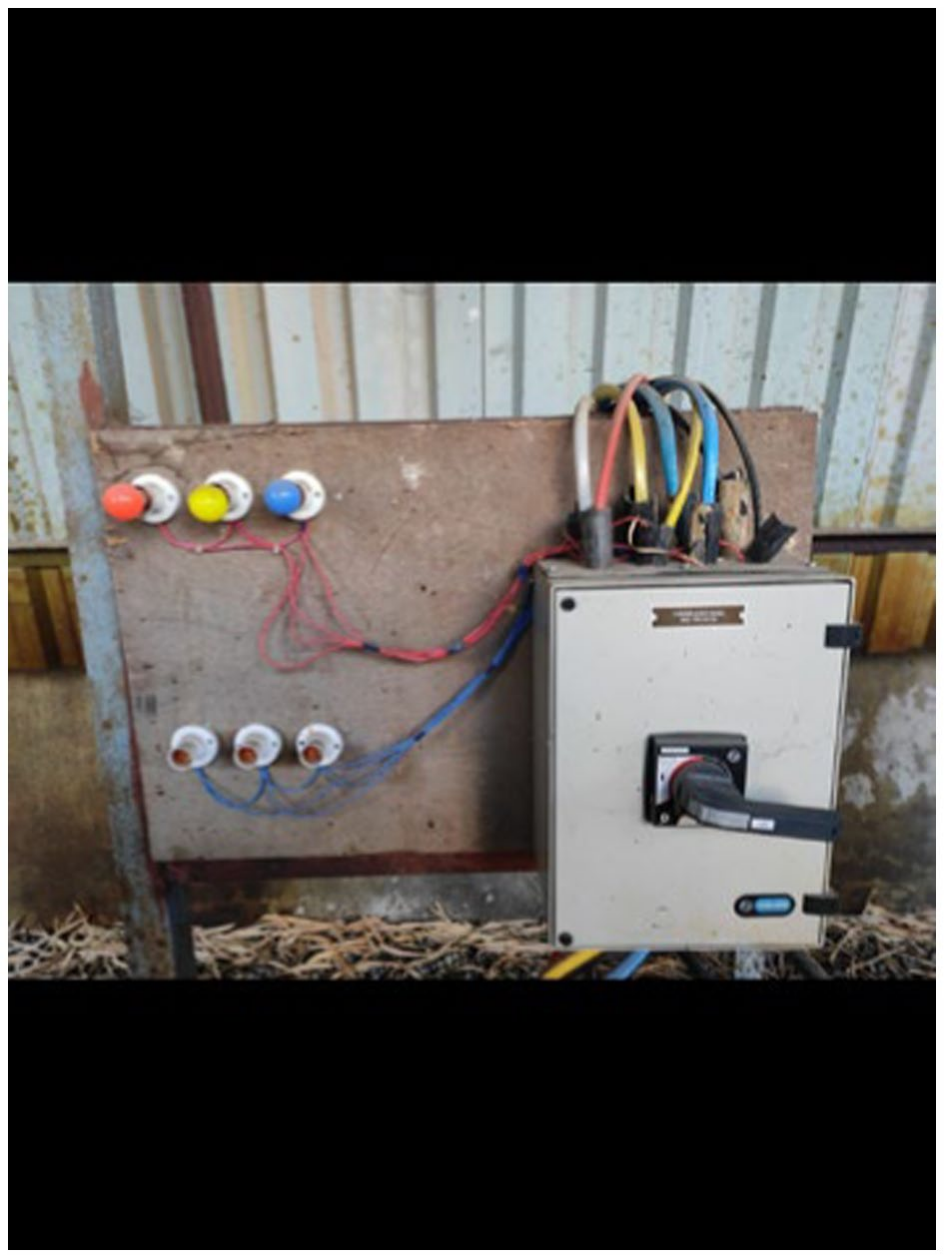
Power Discom main supply line.

**Figure 10:**
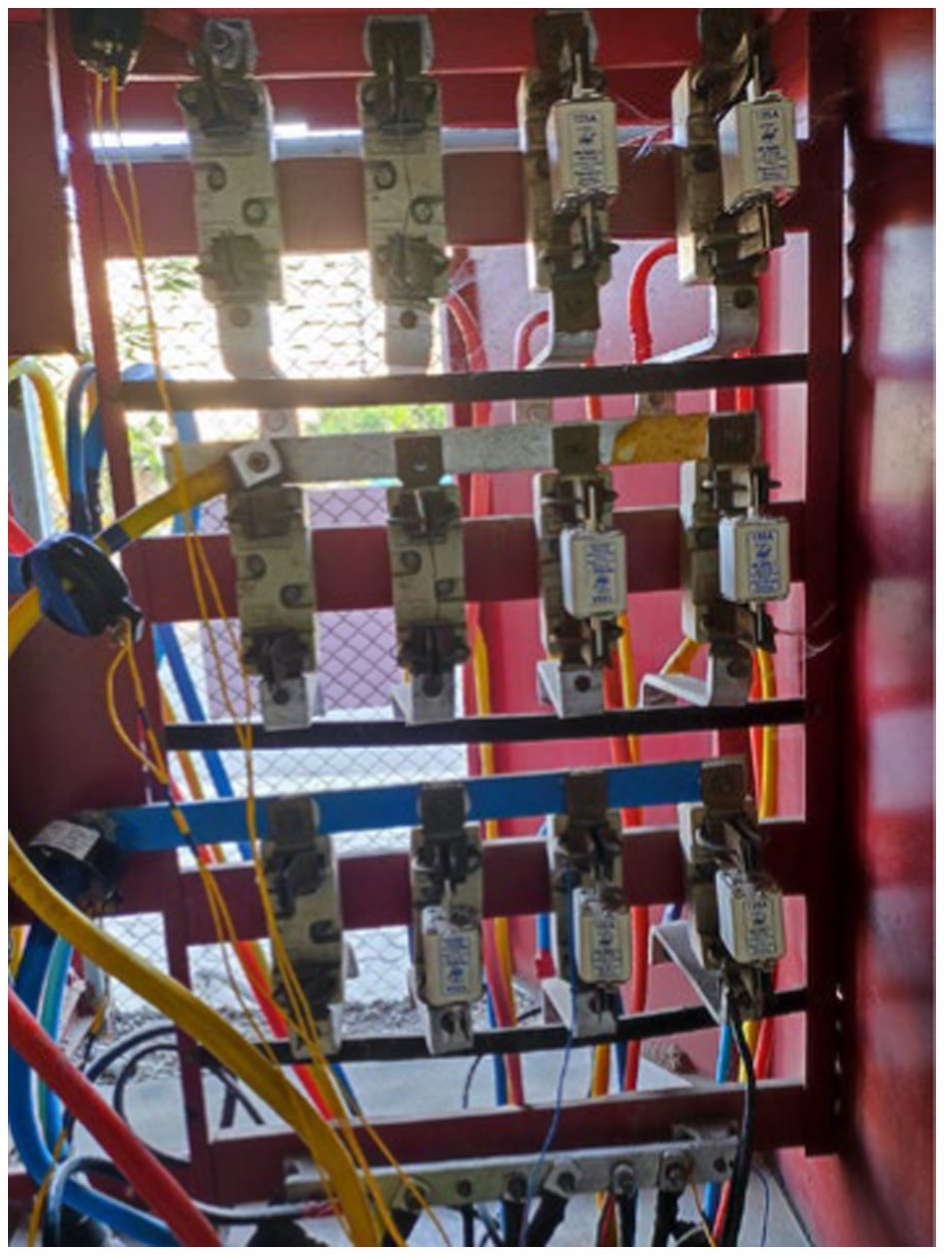
Current transformer.

The USB connector (Figure 8) consolidates the data from the meter and transmits it to the Raspberry Pi via USB. The data collection system consistently monitored various parameters related to electricity consumption at one-minute intervals. The project commenced on December 30, 2021, at 17:38:00 and continued until December 4, 2023, at 04:49:00.

The Raspberry Pi (Figure 6) serves as a microcontroller that manages the storage, retrieval, and uploading of data to Dropbox (a cloud storage service) using an internet connection. An internet connection is provided to the Raspberry Pi through the Wifi modem that operates with a SIM card. The function of the current transformer is to lower the current value for a specified voltage, which facilitates simpler power calculations. However, the heat generated within the main supply line caused the Raspberry Pi’s battery to swell, ultimately shortening its lifespan. This resulted in three instances during the data collection phase where the battery depleted, causing interruptions and resulting in a total loss of 50 min of data.

### 3.2 Data analysis and system implementation

Data analysis and DL modeling were conducted using Visual Studio Code on a laptop equipped with an Intel(R) Core(TM) i5-1035G1 CPU operating at 1.00GHz with a maximum frequency of 1.19 GHz, featuring a 64-bit operating system, a 64-bit processor, 8.00 GB of RAM, and running Windows 11 Home Single Language. The electricity parameters recorded during the monitoring included:

- Timestamp: the exact date and time when the data entry was made.
- Slave ID: unique identifier assigned to a device or sensor reporting the data.
- Total Power (Ptot): the comprehensive power consumption measured in watts.
- Power Phase R: the power usage in the Red (R) phase.
- Power Phase Y: the energy consumed in the Yellow (Y) phase.
- Power Phase B: the power usage in the Blue (B) phase.
- Power Factor Total (PFa): the overall power factor, indicating how effectively the power is utilized.
- Power Factor R: the power factor specific to the R phase.
- Power Factor Y: the power factor associated with the Y phase.
- Power Factor B: the power factor corresponding to the B phase, where a value close to 1 or 100% indicates an efficient conversion of electrical power to usable work.
- Apparent Power Total (St): the total apparent power across all phases, typically measured in volt-amperes (VA).
- Apparent Power R (Sr): apparent power within the R phase.
- Apparent Power Y (Sy): apparent power in the Y phase.
- Apparent Power B (Sb): apparent power measured in the B phase.
- Line Voltage Average (VLa): average voltage recorded across all phases.
- Voltage R to Y (Vry): voltage recorded between the R and Y phases.
- Voltage Y to B (Vyb): voltage measured between the Y and B phases.
- Voltage B to R (Vbr): voltage recorded between the B and R phases.
- Voltage Average (Va): average voltage calculated for each phase.
- Voltage R (Vr): voltage specific to the R phase.
- Voltage Y (Vy): voltage specific to the Y phase.
- Voltage B (Vb): voltage specific to the B phase.
- Current Total (It): total current drawn across all phases, reported in amperes (A).
- Current R (Ir): current level recorded in the R phase.
- Current Y (Iy): current level recorded in the Y phase.
- Current B (Ib): current level recorded in the B phase.
- Frequency: the frequency of the alternating current (AC) power supply, typically measured in hertz (Hz), usually around 50 or 60 Hz.
- Energy: total energy consumption measured in kilowatt-hours (kWh), recognizing energy usage over a specified period.
- Volt-amperes hour (Vah): this measures apparent power usage over time, akin to kWh but reflects total apparent power rather than merely real power.

Analysis of the collected data revealed a seasonal trend in electricity consumption that peaks in January and declines in May, followed by another increase from July through November/December. This fluctuation in consumption is attributed to the two academic semesters: July to December and January to June. The pattern indicates that energy consumption correspondingly rises with academic activities, gradually increasing at the beginning of each semester as labs and computer systems come into operation. Typically, electricity usage escalates post 9 AM when campus facilities open and decreases around 6 PM when operations cease. Data collection was conducted up until October 2023.

### 3.3 Hybrid neuro-fuzzy DL model

**Figure 11:**
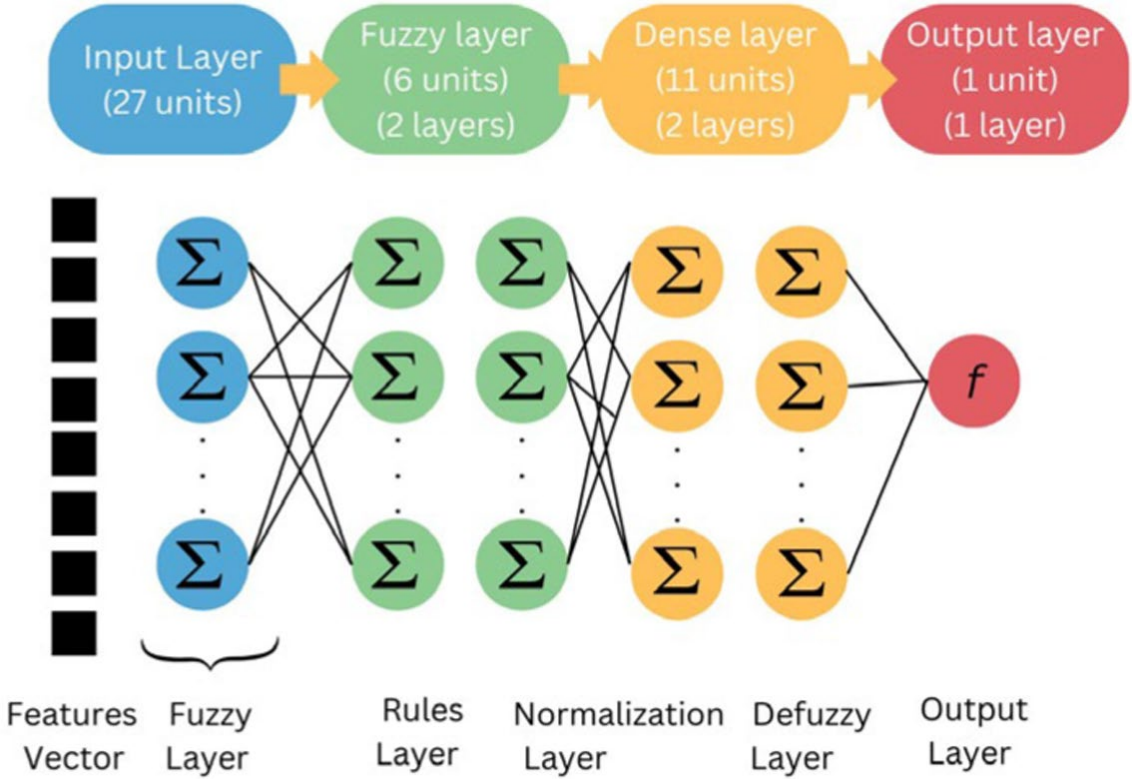
Hybrid neuro-fuzzy DL model.

The Hybrid DL-IoT BEMS backbone was built with a hybrid Neuro-fuzzy DL model. The hybrid Neuro-fuzzy DL model includes a custom Fuzzy Layer, a hidden layer, and an output layer (Figure 11).

It takes energy parameters as input and predicts energy output as follows. Let X ∈ R^*N* × 2^ be the input matrix, where *N* is the number of samples, and each sample contains two energy parameters, *x*_1_ and *x*_2_. The model is composed of the following layers:

Fuzzy Layer: This layer performs a fuzzy transformation on the input. It can be described by the following equation:


$$Z=\sigma \left(X\cdot W_{f}\right)$$

Where W*_f_* ∈ R^2 × *M*^ is the weight matrix of the Fuzzy Layer (with *M* being the number of output neurons in this layer), and *σ* represents the sigmoid activation function applied element-wise.

Hidden Layer(s): The output from the Fuzzy Layer is then processed by a fully connected hidden layer(s), where the activation function is ReLU (Rectified Linear Unit):


$$H=\mathrm{ReLU}\left(Z\cdot W_{h}+b_{h}\right)$$

Where W*_h_* ∈ R^*M* × *P*^ is the weight matrix of the hidden layer, *P* is the number of neurons in this layer, and b*_h_* is the bias vector for the hidden layer.

Output layer: finally, the output layer produces the energy prediction:


$$yˆ=H\cdot W_{o}+b_{o}$$

Where ˆ*y* ∈ R is the predicted energy output, W*_o_* ∈ R^*P* × 1^ is the weight matrix for the output layer, and b*_o_* is the output bias.

Loss function: the model is trained using the Mean Squared Error (MSE) loss function defined as follows:

$MSE=\frac{1}{N}\overset{N}{\underset{i=1}{\sum }}{\left(y_{i}-{\hat{y}}_{i}\right)}^{2}$

Where *y_i_* represents the true energy output for sample *i* and ˆ*y_i_* denotes the predicted output (Algorithm 1).

**ALGORITHM 1:**
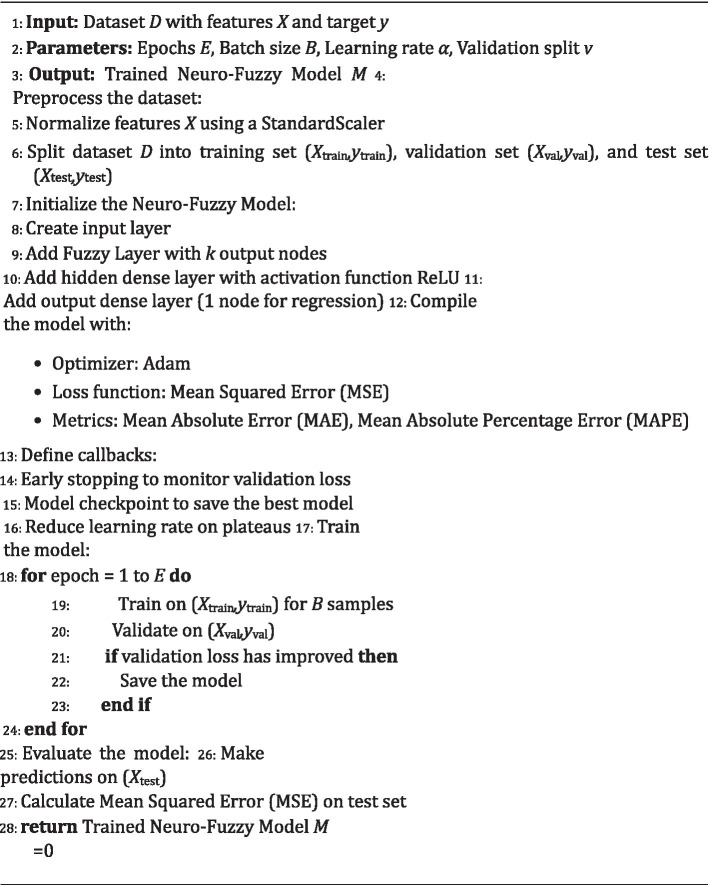
Hybrid neuro-fuzzy DL model based BEMS.

Further, the Neuro-Fuzzy Model was trained over electrical data collected over a period of 2 years through on premise monitoring in a university campus.

## 4 Experimental work

### 4.1 Statistical feature analysis using *t*-test

Initially the ordinary least square (OLS) model from python 3 stats models library was used to understand which feature has a statistically significant (*p* ≤ 0.05). Tables 1, 2 gives the results of the model.

**Table 1:** OLS regression results.

	Coefficient	Std. error	t-statistic	*p* ≤ 0.05
const	2.68e+04	369.892	72.446	0.000
Ptot	−5.223	0.754	−6.929	0.000
Pr	4.989	0.767	6.504	0.000
Py	−34.042	1.106	−30.786	0.000
Pb	23.829	2.356	10.115	0.000
PFa	−31.792	1.843	−17.250	0.000
PFr	31.988	1.976	16.192	0.000
PFy	28.112	2.203	12.762	0.000
PFb	−91.892	5.538	−16.593	0.000
St	81.890	1.646	49.751	0.000
Sr	−81.856	1.647	−49.700	0.000
Sy	−360.209	24.784	−75.301	0.000
Sb	523.955	25.157	101.606	0.000
Va	440.008	174.416	2.523	0.012
Vr	−208.909	58.139	−3.593	0.000
Vy	−228.410	58.140	−3.929	0.000
Vb	−274.984	58.143	−4.729	0.000
It	−19.649	0.365	−53.846	0.000
Ir	19.673	0.365	53.886	0.000
Iy	100.629	1.200	83.867	0.000
Ib	−139.951	1.209	−115.778	0.000
freq	−411.549	7.454	−55.214	0.000
VAh	0.951	1.53e-05	6.23e+04	0.000

**Table 2:** Performance metrics for different BEMS and the Hybrid DL-IoT BEMS.

Model	MAE	MSE	R2
R. Sanchez et al.	3.86e^2^	2.2e^3^	9.6e^−1^
J. Kim et al.	4e^2^	2.34e^3^	9e^−1^
E. Bicici	4.01e^2^	2.27e^3^	8.9e^−1^
Proposed method	3.85e^2^	2.21e^3^	9.9e^−1^

From the above table, the OLS regression model for predicting “Energy” shows a perfect fit with an R-squared of 1.000, though this may indicate overfitting given the high F-statistic of 2.232e+08 and condition number of 1.09e+16, suggesting multicollinearity. Most coefficients are statistically significant (*p* ≤ 0.05), indicating strong relationships with “Energy.” Diagnostic tests show non-normal residuals and positive autocorrelation (Durbin-Watson = 0.048). The model includes 22 predictors with 658,006 observations. While the high *R*^2^ and low AIC/BIC suggest a good fit, further validation and addressing multicollinearity and residual issues are recommended for robustness.

### 4.2 Results

The performance of the proposed hybrid Neuro-fuzzy deep learning BEMS model is assessed and contrasted using three widely recognized metrics. The first metric is the Mean Absolute Error (MAE), computed using Equation 1, which offers a simple indication of the average absolute deviations.


(1) $$MAE=\frac{1}{n}\overset{n}{\underset{i=1}{\sum }}|y_{i}-{\hat{y}}_{i}|$$

The second metric is the Mean Squared Error (MSE), determined using Equation 2, which places greater emphasis on larger errors by squaring the differences.


(2) $$MSE=\frac{1}{n}\overset{n}{\underset{i=1}{\sum }}{\left(y_{i}-{\hat{y}}_{i}\right)}^{2}$$

Lastly, the Coefficient of Determination (*R*^2^) is used, as shown in Equation 3, to quantify the amount of variance in the outcome that the model accounts for.


(3) $$R^{2}=1-\frac{\sum_{i=1}^{n}{\left(y_{i}-{\hat{y}}_{i}\right)}^{2}}{\sum_{i=1}^{n}{\left(y_{i}-\bar{y}\right)}^{2}}$$

In these Equations 1–3, *y_i_* denotes the actual values, ˆ*y_i_* signifies the values predicted by the model, and ¯*y* represents the average of the actual values. Analyzing MAE, MSE, and *R*^2^ together provides a thorough evaluation of the model’s effectiveness.

### 4.3 Discussion

**Figure 12:**
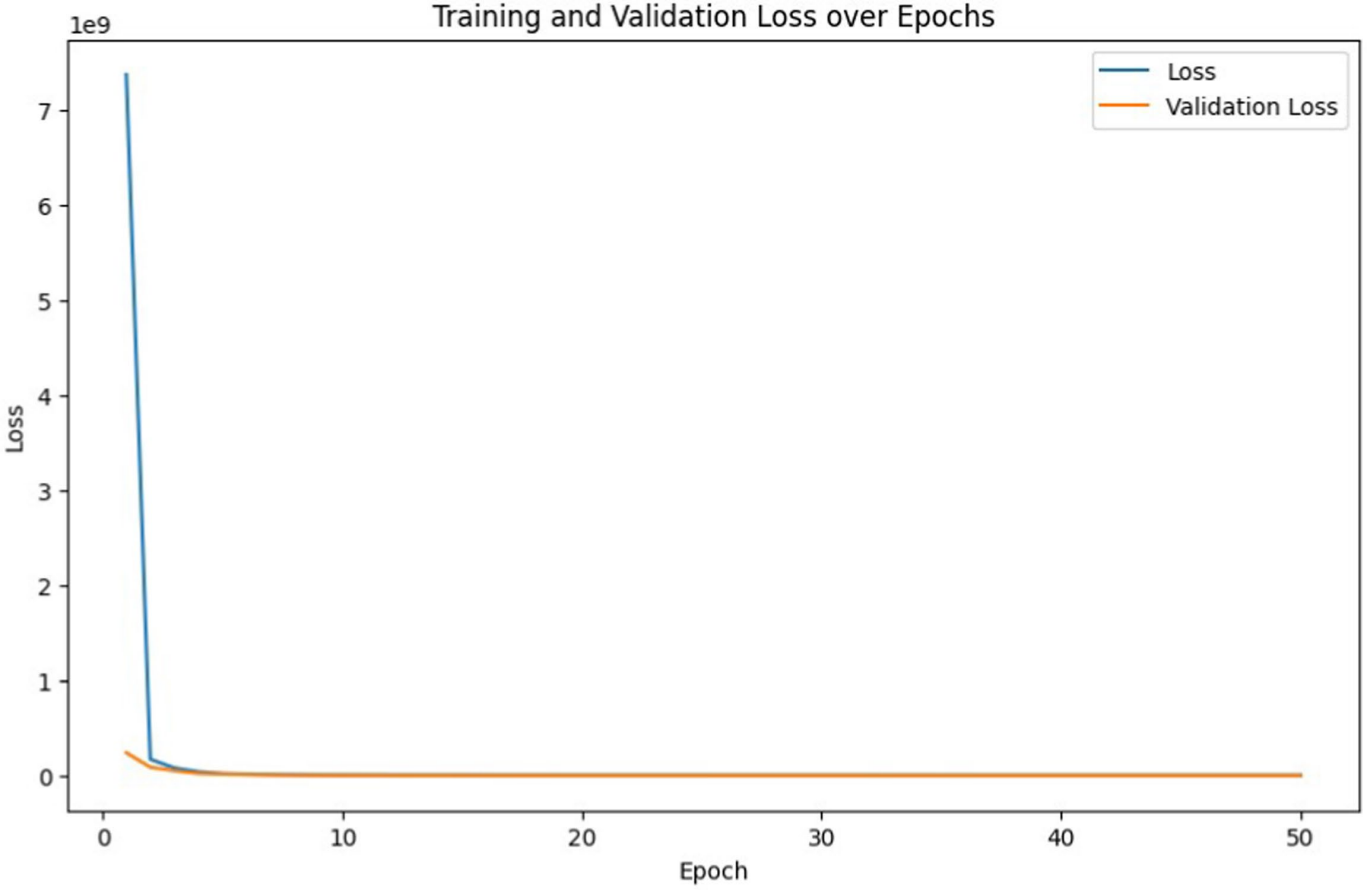
Training set loss and validation set loss with epochs for deep learning based BEMS.

Figure 12 illustrates the progression of training set loss and validation set loss across the specified epochs during the training of our deep learning model for Building Energy Management Systems (BEMS).

**Figure 13:**
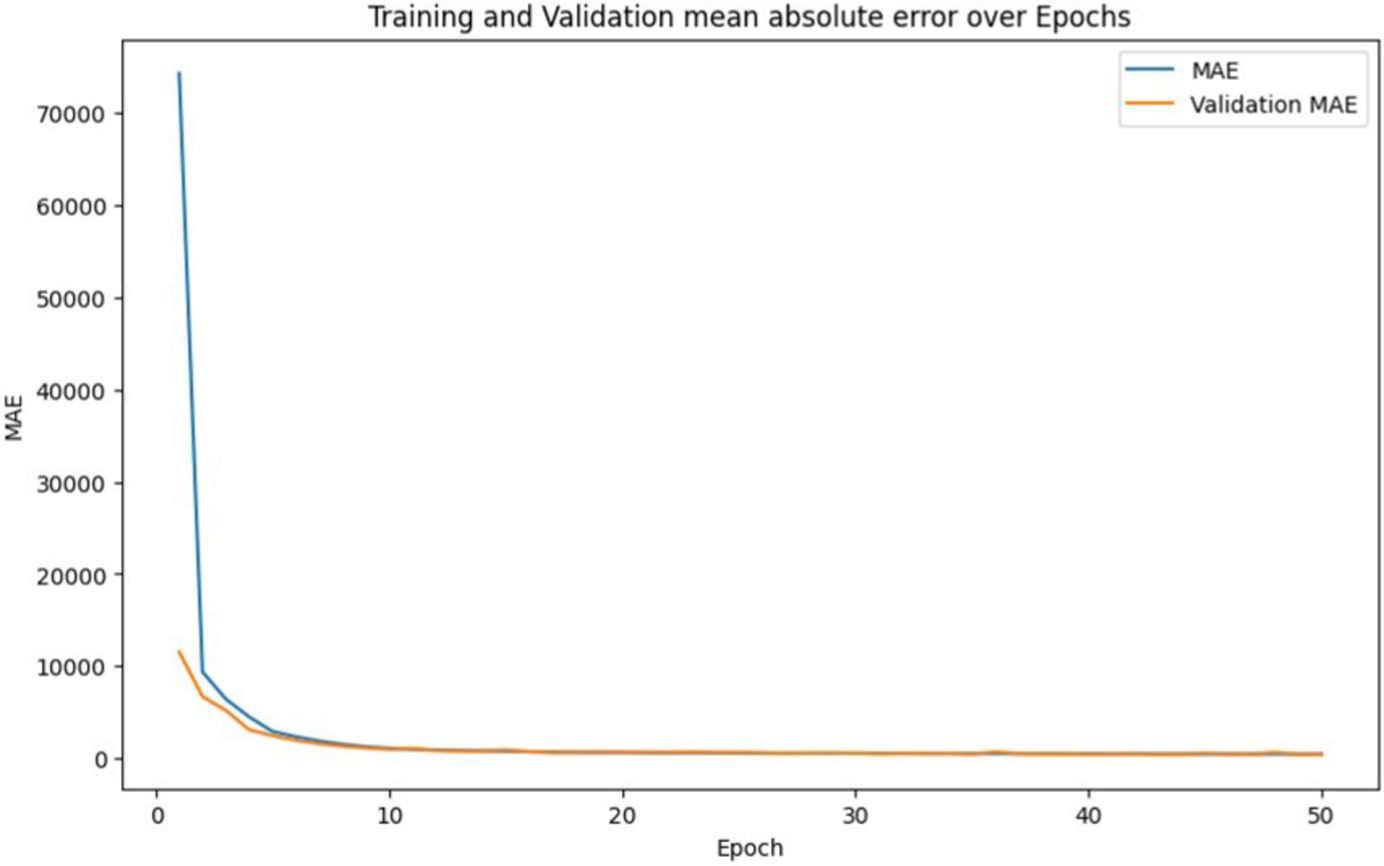
Training set mean absolute error and validation set mean absolute error with epochs for deep learning based BEMS.

From the initial epoch, we observe a significant decrease in both training loss and validation loss, indicating effective learning by the model. At epoch one, the training loss began at a substantial ∼7.38 × 10^9^ and was reduced to approximately 503881.28 by the end of epoch 50, showcasing the model’s ability to generalize and learn the underlying patterns in the training data. In Figure 13, we observe the progression of both the training and validation mean absolute error (MAE) over the epochs. Initially, at the first epoch, the training MAE is considerably high at approximately 74315.91, indicating that the model is not yet well-tuned to the dataset. However, as training progresses through multiple epochs, there is a noticeable decline in both training and validation MAE, suggesting that the model is effectively learning from the data.

**Figure 14:**
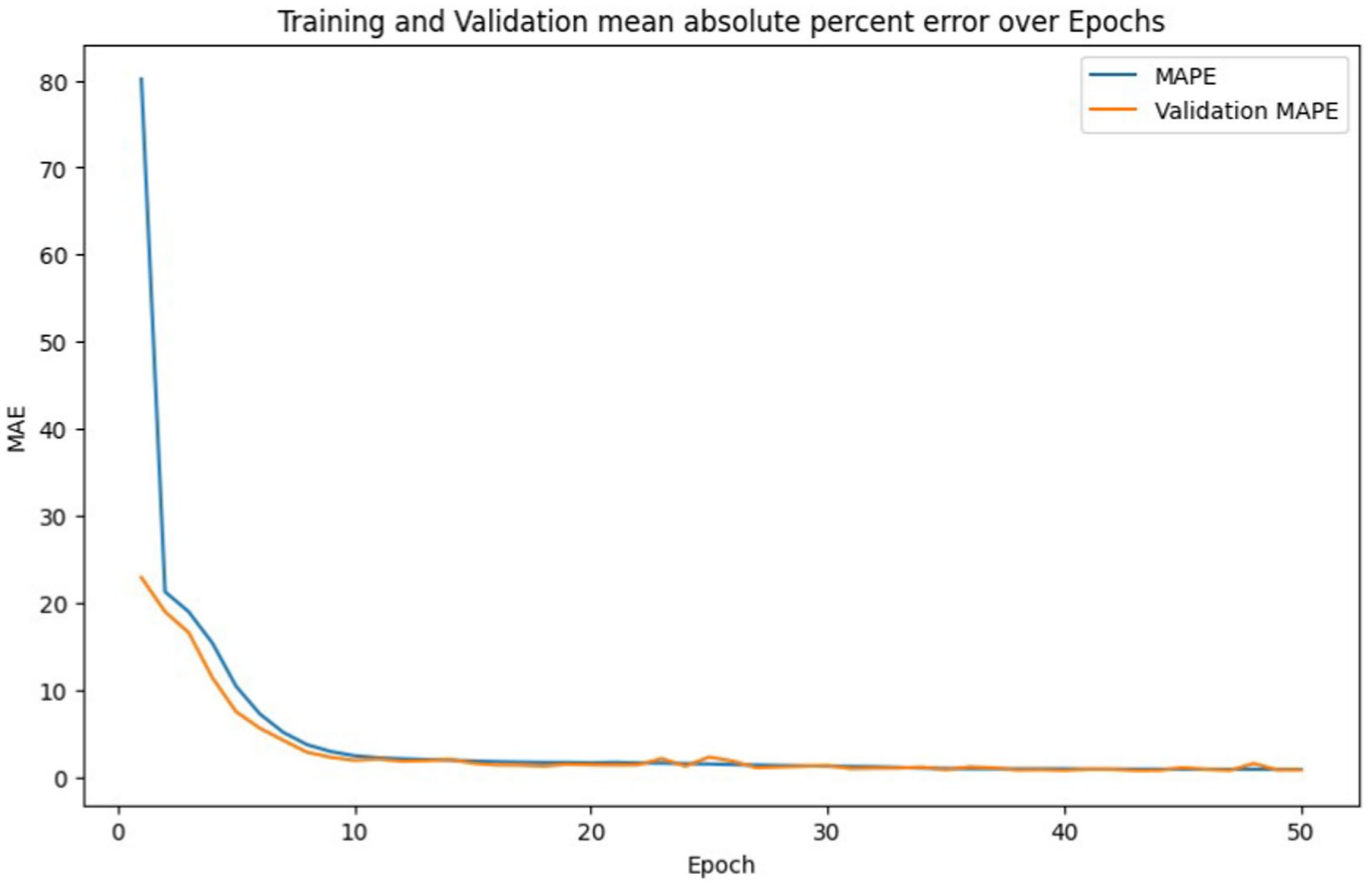
Training set mean squared error and validation set mean squared error with epochs for deep learning based BEMS.

By the end of epoch 50, the training MAE reaches approximately 459.19, while the validation MAE is reported at around 436.42. This relatively low error indicates that the model has achieved substantial accuracy, maintaining a good balance between training and validation error. Such convergence of training and validation MAE suggests that the model is not overfitting and can generalize well to unseen data. This represents a promising outcome for the application of deep learning techniques in optimizing Building Energy Management Systems (BEMS). As illustrated in Figure 14, the MAPE shows a consistent decline throughout the training process, indicating an improvement in the model’s predictive accuracy.

**Figure 15:**
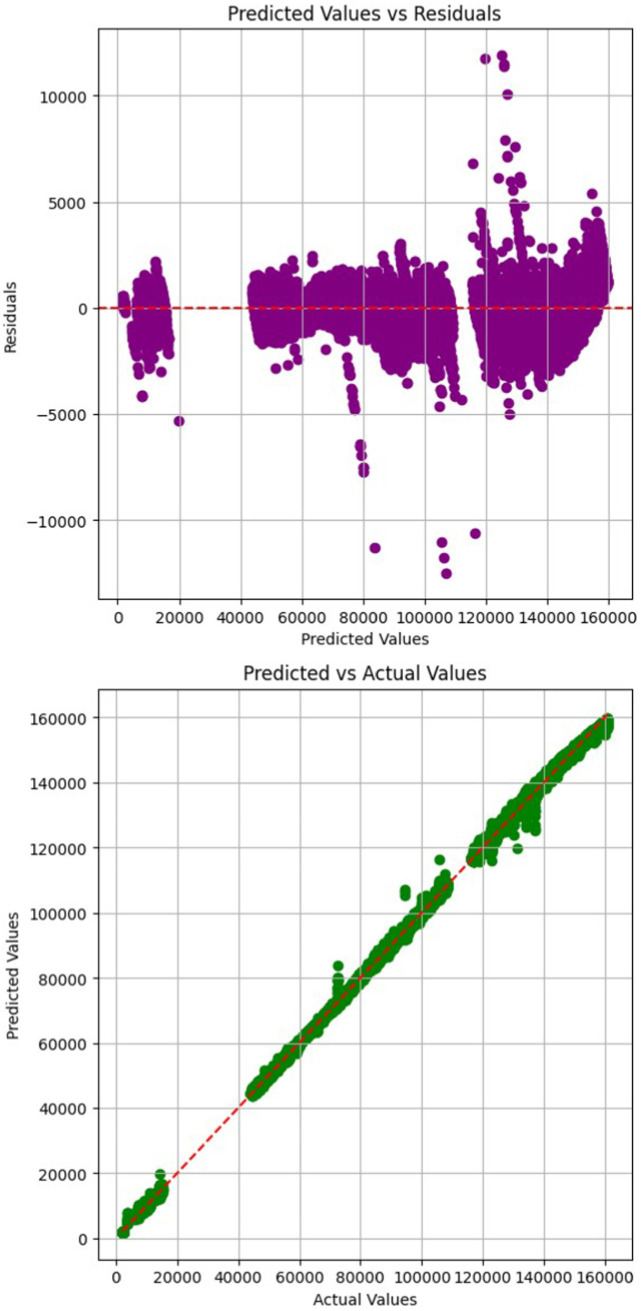
Proposed model: prediction on test set (residual plots).

Figure 15 presents a scatter plot that depicts the alignment between predicted values (on the y-axis) and actual values (on the x-axis). A second plot is Predicted values (on the x-axis) vs. Residuals (on the y-axis). In predicted vs. residuals plot, it is important to ascertain if the residuals are distributed randomly along the red dotted line. Absence of patterns indicate that the model is able to learn the variability in the data effectively. The predicted values from the proposed model are observed to be closely aligned with the red line, indicating a strong correlation.

**Figure 16:**
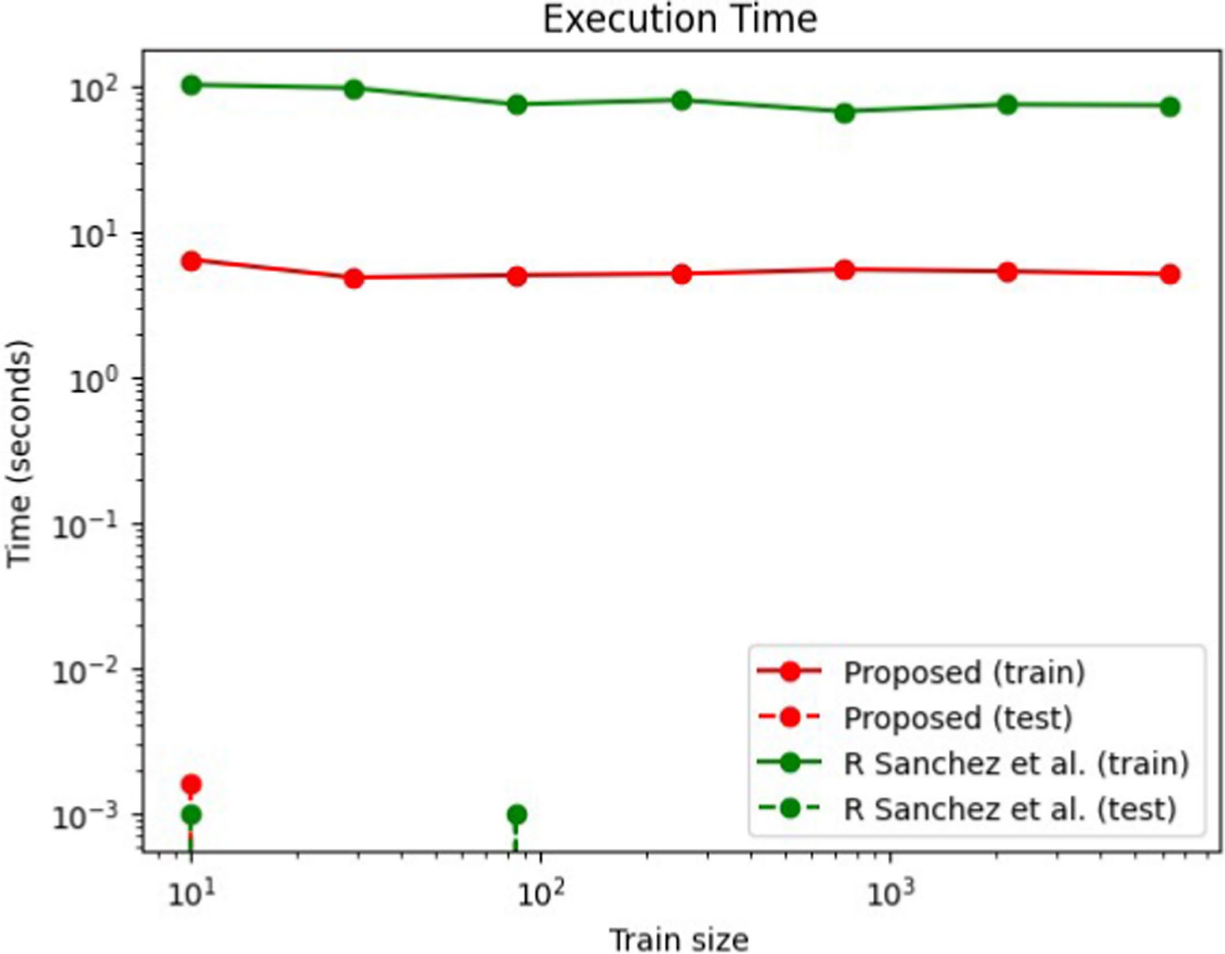
Hybrid DL-IoT BEMS: time vs. train size.

Figure 16 provides a comparison of the fitting and prediction times of the proposed model against the BEMS by Martınez-Sanchez et al. (2023) evaluated across various training set sizes. When the training size is increased exponentially the time needed for training is monitored to growth. If the time needed is also growing exponentially then the model is not scalable for large datasets. As seen from the image, as the train dataset increases in size the training time is linear and so the model is scalable for large datasets too. In terms of fitting time, the model developed by Martınez-Sanchez et al. (2023) for Building Energy Management Systems (BEMS) demonstrates faster performance for medium-sized training sets (up to a few thousand samples).

However, the hybrid Deep Learning-Fuzzy model in our proposed approach shows improved scalability for larger datasets. Regarding the prediction time, the proposed model generally exhibits faster performance compared to Lasso across all training set sizes due to its learned sparse solution. Nevertheless, practical outcomes may vary due to specific implementation aspects within the Keras-tensor flow library, where both models are integrated. Both our proposed model and Martınez-Sanchez et al. (2023) method effectively minimize the MSE, with our approach achieving this goal using a smaller training dataset.

Furthermore, as presented in Table 2, the performance of the proposed method against established metrics is superior when compared to other BEMS approaches documented in existing literature. The MAE, MSE should be as low as possible which would indicate the predicted values for energy consumption are as close to the actual values. The *R*^2^ is close to 1 which proves that the model is generalizing to out-of-sample data. Tables 3, 4 gives the qualitative analysis of proposed methods with state of the art on AI BEMS features and capabilities. A gap exists in studies that integrate both hardware-based (Arduino Uno and sensors) and data-driven (ML/DL and historical data) approaches, suggesting opportunities for hybrid frameworks that combine real-time monitoring with predictive analytics. Additionally, cost optimization is addressed across multiple studies, particularly in those involving energy sensors or data-driven methods, yet there is limited research on optimizing consumption through wireless-enabled solutions. These findings underscore the need for further exploration into integrated, adaptive, and scalable energy monitoring frameworks that leverage both sensor-based real-time data acquisition and ML-driven predictive analytics for comprehensive energy management.

**Table 3:** Qualitative analysis of proposed methods with state of the art on AI BEMS features.

References	IoT	BEMS DL Techniques	Energy Sensors	Cost Optimization
Nabavi et al. (2021)	✗	✗	✗	✗
Abu-Shikhah et al. (2011)	✗	✗	✗	✓
Adepoju et al. (2007)	✓	✗	✓	✓
Liu et al. (2019)	✗	✓	✗	✓
Al-Anbuky and Bataineh (1995)	✓	✗	✓	✓
Moutaib et al. (2022)	✓	✗	✓	✓
Amjady (2002)	✗	✗	✗	✗
Elashry et al. (2024)	✗	✓	✗	✓
Madhav and Sunil, 2022	✗	✓	✗	✗
Okolobah and Ismail (2013)	✗	✓	✗	✗
Montgomery et al. (1990)	✗	✓	✗	✗
Yildiz et al. (2017)	✗	✓	✗	✗
Saravanan et al. (2012)	✗	✓	✗	✗
Proposed model	✓	✓	✓	✓

**Table 4:** Qualitative analysis of proposed methods with state of the art on AI BEMS capabilities.

References	Wireless capability	Historical data usage
Nabavi et al. (2021)	✗	✗
Abu-Shikhah et al. (2011)	✗	✗
Adepoju et al. (2007)	✗	✗
Liu et al. (2019)	✗	✓
Al-Anbuky and Bataineh (1995)	✗	✗
Moutaib et al. (2022)	✗	✗
Amjady (2002)	✗	✗
Elashry et al. (2024)	✗	✓
Madhav and Sunil, 2022	✗	✓
Okolobah and Ismail (2013)	✗	✗
Montgomery et al. (1990)	✗	✓
Yildiz et al. (2017)	✗	✓
Saravanan et al. (2012)	✗	✓
Proposed model	✓	✓

### 4.4 Cost and schedule optimization

**Figure 17:**
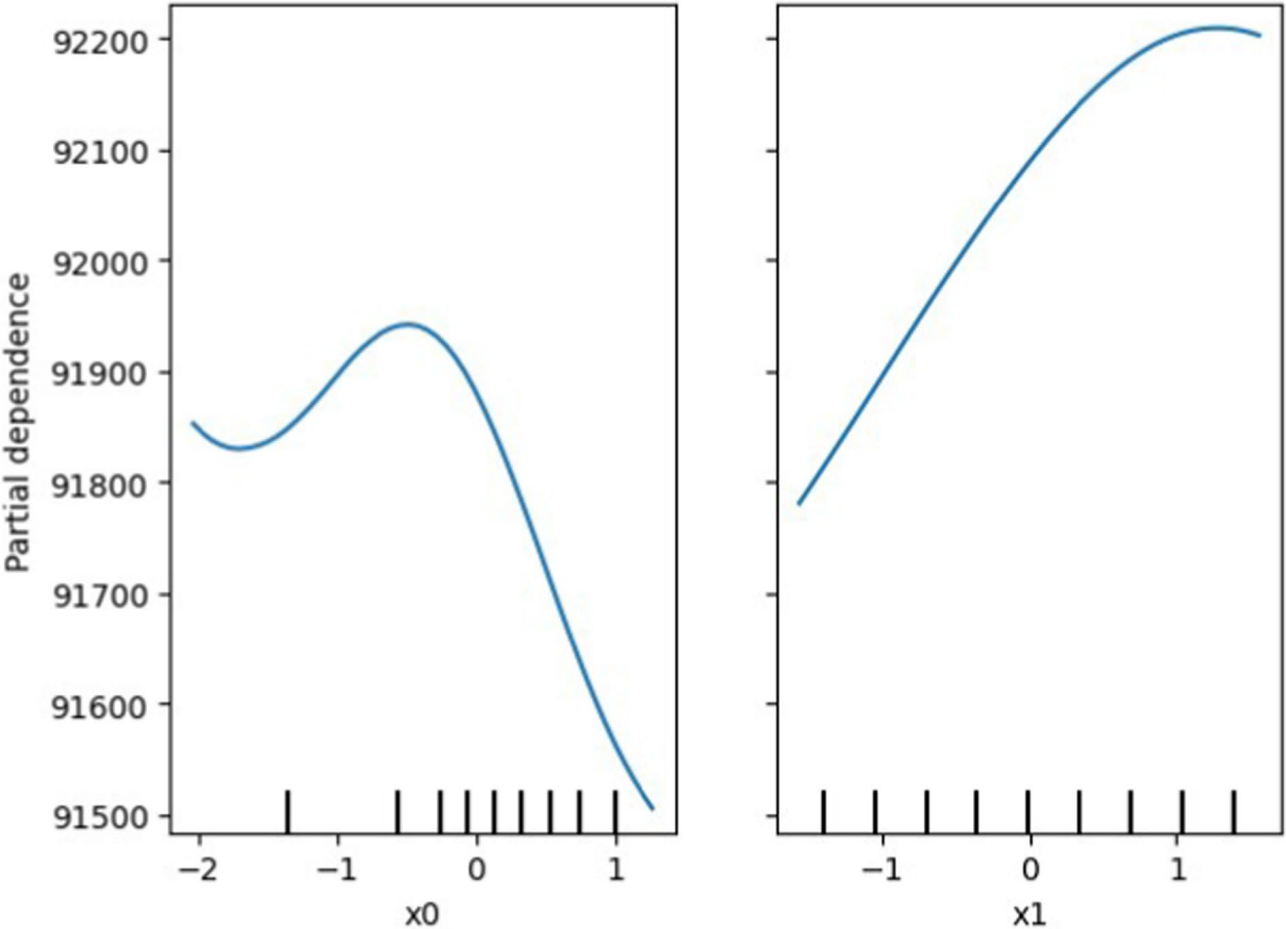
Hybrid DL-IoT BEMS: partial dependence of Ptot (x0) and PFr (x1) on energy consumption.

By utilizing the proposed model, one can identify the primary areas for potential energy savings by organizing the high energy consumption activities of the university such as workshops, lab sessions and experimental work. For this purpose, the existing activities at the institute were studied for the period January 2024 to March 2024 and their energy consumption was noted. Further using the hybrid neuro-fuzzy deep learning model a partial dependence plot was completed, as shown in Figure 17.

It serves as a helpful technique to illustrate the relationship between input features and the output feature, while accounting for the influence of other input variables. Once the model is trained, it is deployed for model inference, enabling real-time predictions of energy demand. To enhance energy efficiency, the system integrates the IoT-based BEMS with an L-BFGS-based energy optimizer. The Limited-memory Broyden-Fletcher-Goldfarb-Shanno (L-BFGS) algorithm is employed to optimize energy usage by minimizing wasteful consumption while maintaining operational efficiency. The findings indicate that Ptot shows an inverse correlation and PFr shows a direct correlation with energy consumption. Therefore, changing these parameters beyond their current values could lead to significant reduction in energy and cost reductions. An enhancement of one unit in Ptot corresponds to a decrease in 5 units in energy consumption, while a similar increase in PFr results in a 27 unit increase in energy consumption.

**Figure 18:**
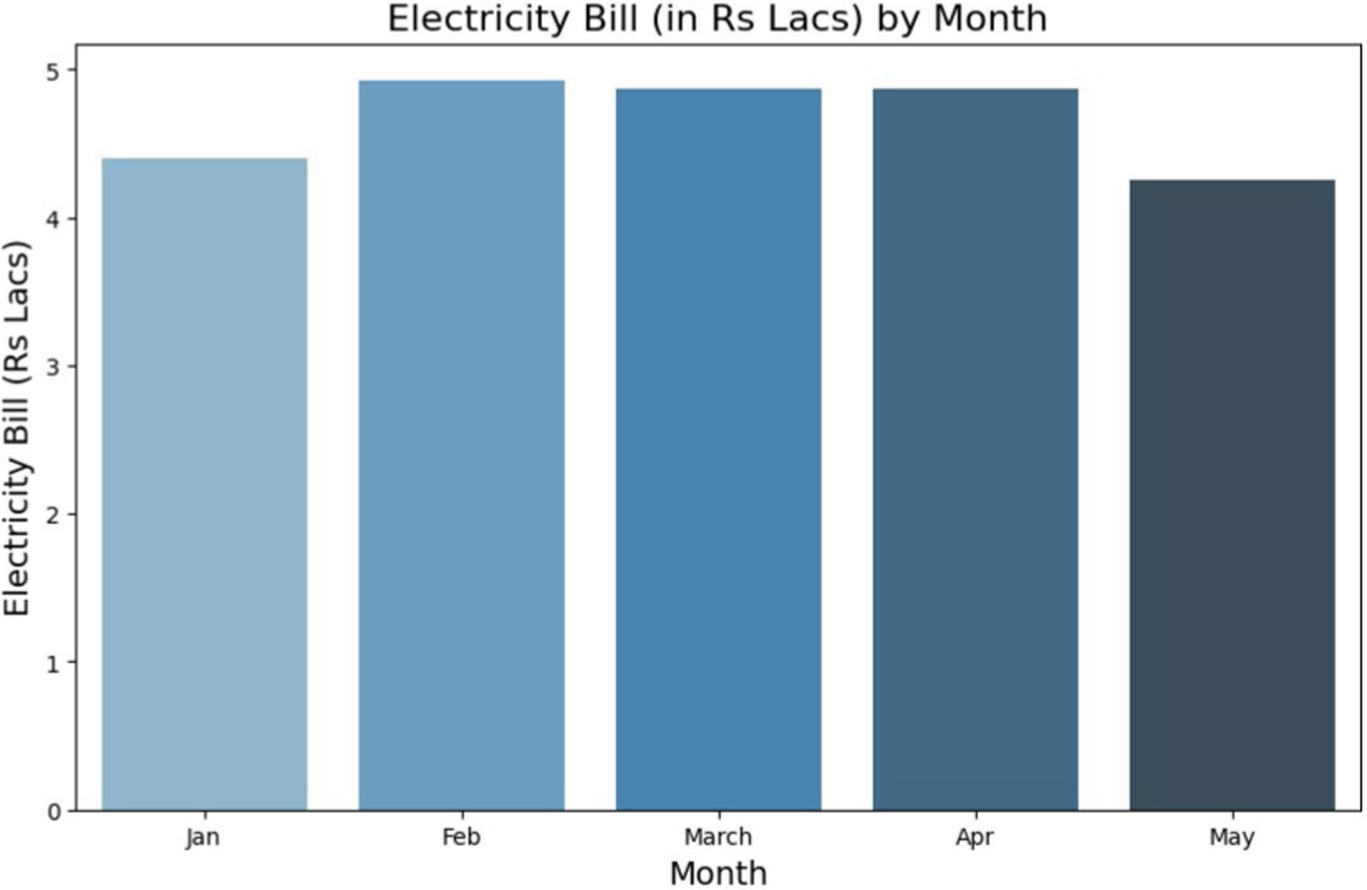
Cost and schedule optimization using hybrid DL-IoT BEMS results: reduction in electricity consumption.

To achieve the reduction in Ptot and PFr to optimum levels, the existing activities were studied for the period of January and February 2024 and the Hybrid DL-IoT BEMS was deployed as a web portal on streamlit cloud platform for daily monitoring. The impact was visible from months of March, April and May in form of reduction in consumption of electricity bill (Figure 18). In the 3 months if the consumption of January and February was extrapolated by increasing the February electricity bill by the 10, 20, 30% for the months of March, April and May, respectively, (as was seen in the previous year 2023’s electricity bill) and assuming no reduction in consumption then the energy costs for March, April and May cumulative would have been Indian rupees (INR) 17.39 lacs and the actual consumption was INR 14 lacs resulting in a savings on INR 3.39 lacs.

## 5 Concluding remarks and future works

In conclusion, the main insights from this study suggest that employing specific features such as power factor, apparent power, phase-wise voltage, phase-wise current, and frequency can lead to a highly precise model for energy consumption predictions. By developing a fuzzy layer to the ANN architecture and building a hybrid neuro-fuzzy DL model based BEMS the electricity consumption data was accurately modeled. The required equipment consists of commonly accessible open-source hardware such as raspberry pi, multifunction meter, USB connectors and WiFi modules. Moreover, the open-source software tools such as keras-tensor flow module of python programming language and data preparation and preprocessing modules of scikit-learn toolbox were found to be suitable for developing data-driven models. Further training the hybrid neuro-fuzzy deep learning model does not necessitate extensive datasets, provided that the features are both intuitive and informative.

## Data Availability

The datasets presented in this study can be found in online repositories. The names of the repository/repositories and accession number(s) can be found in the article/supplementary material.
